# Case report: vertebral osteomyelitis secondary to a dental abscess

**DOI:** 10.1186/s12879-020-4857-7

**Published:** 2020-02-12

**Authors:** Abhijith Bathini, Christina R. Maxwell, Hirad Hedayat, James Barrett, Zakaria Hakma

**Affiliations:** 1 Drexel University College of Medicine, 219 N Broad St 7th flr Philadelphia, Philadelphia, PA 19107 USA; 2 Global Neurosciences Institute, Lawrenceville, NJ USA

**Keywords:** Vertebral osteomyelitis, Veillonella, Neurosurgery

## Abstract

**Background:**

Vertebral osteomyelitis can be attributed to many factors including immunosuppression, diabetes, malignancy, collagen disease, periodontal disease, open fractures, and endoscopic procedures. Anaerobic bacteria, such as Veillonella species, are found in the oral cavity and are rarely implicated in the infection. This report describes vertebral osteomyelitis secondary to a dental abscess with positive Veillonella cultures.

**Case Description:**

A 76-year-old man presented to the hospital due to back pain with a four-day history of fever and chills. CT scans revealed several abscesses in the lumbar region as well as indications of vertebral osteomyelitis. After a psoas drain, the patient began antibiotics with a combination of ampicillin-sulbactam, metronidazole, and levofloxacin, but due to the patient’s penicillin allergy, he was initially desensitized to this antibiotic for a significant period of time. Laminectomies, foraminotomies, and facetectomies were performed, but the infection spread to vertebral levels. The patient was then switched to a combination of vancomycin, metronidazole, and levofloxacin which eliminated the infection. Final laminectomy was performed with posterior segmental instrumentation and arthrodesis. Post-operatively, there were no signs of infection. The patient recovered well and regained mobility. Deeper examination of the patient’s medical history revealed a severe tooth abscess immediately before the onset of bacteremia.

**Conclusion:**

We believe that a delay in the onset of antibiotic treatment is what led to the initial bacteremia that ultimately took root in the lower lumbar vertebrae. To the best of our ability, we could identify only one other case that linked vertebral osteomyelitis to the oral cavity.

## Background

Vertebral osteomyelitis is a bacterial infection of the bones of the spine resulting from hematogenous spread, direct inoculation, or contiguous spread from soft tissue infection [1, 2]. It is most often encountered at the lumbar levels, followed by thoracic and cervical levels [1–3]. The vast majority of bacterial species implicated in this infection are facultative anaerobes. The most common gram-positive species are *Staphylococcus aureus*, *Staphylococcus epidermidis, Streptococcus* species, and *Enterococcus* species while the most common gram-negative species are *E. coli*, *Pseudomonas aeruginosa,* and *Proteus mirabilis.*^4,5,6,7^ However, vertebral osteomyelitis is rarely caused by strictly anaerobic organisms, accounting for only 3–5% of all spondylodiscitis cases [4–6].

*Veillonella* is an anaerobic gram-negative bacterial species known to be found in the oral cavity, gastrointestinal tract, and female genital tract [4, 7–9]. Thirteen species of the genus have been identified, with only six having been isolated in human oral cavities [10]. Risk factors for *Veillonella* infections are typically immunosuppression, diabetes, malignancy, collagen disease, periodontal disease, open fractures, and instrumentation with endoscopy. Infections can also occur without any particular risk factors in healthy individuals. The most common ones are bone and joint infections, followed by endocarditis and bacteremia [3, 4, 8–12].

This case report received an exempt review status from the institutional review board and written consent was obtained from the patient allowing this information to be published.

## Case presentation

### Initial case presentation

A 76-year-old male diagnosed with *Veillonella* bacteremia presented with a sudden onset of back pain, fever, and chills of four days duration. Although the fever and chills had since subsided, the back pain continued to persist and was exacerbated with positional change. CT scans revealed a psoas abscess with extradural extension, along with indications of lumbar osteomyelitis. MRI scans were unable to be obtained owing to the patient’s pacemaker. Due to medication allergies and growing sensitivity to the infection, the patient was transferred to MICU for a short period to be desensitized to ampicillin-sulbactam. Interventional Radiology then placed a retroperitoneal drain for the psoas abscess as well as a PICC line for IV antibiotic administration at home.

### First surgical operation

During the following week, the Neurosurgery service performed laminectomy at levels L4-S1. Bilateral medial facetectomies and foraminotomies were also performed at L4-L5 and L5-S1 to fully decompress the exiting nerve root and thecal sac. Significant facet arthropathy was noted. The extradural abscess, which was revealed to be a large synovial cyst with no pus, was resected. Cultures were taken and sent for laboratory analysis, revealing the presence of *Veillonella* species. Post-operatively, CT scans indicated a resolving abscess. The patient was discharged and instructed to complete his course of ampicillin-sulbactam at home and switch to metronidazole and levofloxacin for a total six-week course of antibiotics.

### Second presentation

The patient re-presented to the hospital 18 days after the neurosurgical operation complaining of worsening back pain radiating into his posterior buttocks that began 10 days after the operation. CT scans revealed considerable interval decrease in the psoas abscess but extensive osseous destruction in the L5-S1 region, anterior to the laminectomy sites, thereby advancing concerns of osteomyelitis. CT scans revealed trace retrolisthesis of L2 on L3 and L3 on L4, as well as grade 1 anterolisthesis of L4 on L5. There was multilevel disc height loss, most severe at L3-L4 and L5-S1, and persistent narrowing of the thecal sac. Fluid was observed posterior to the surgical site along with phlegmonous changes extending from L4-S1. A small abscess was also seen in the S1 prevertebral soft tissues. Interval erosions of the L5 and S1 endplates were noted, compatible with discitis. All of these findings were consistent with progressive osteomyelitis. Furthermore, bone and tissue cultures revealed continued *Veillonella* infection, perhaps due to a combination of both insufficient debridement as well as inadequate duration of antibiotic treatment. The patient was instructed to begin IV vancomycin in addition to metronidazole and levofloxacin for an additional 6 weeks.

### Third presentation

The patient presented to the office 25 days later with unbearable, radiating lower back pain forcing him to use a wheelchair to move about. CT scans indicated progressive destructive changes of the L5-S1 vertebral body endplates. Two presacral abscesses measuring 4 mm and 7 mm were identified anterior to S1; additional microabscesses were found in the prevertebral phlegmon at the L5 level. A larger abscess measuring 15 mm was identified dorsal to the thecal sac at the previous L5 laminectomy site, leading to a narrowing of the dural sac. Progressive destruction of the vertebral bodies was noted with fragmentation of the inferior endplate of L5 and superior endplate of S1. Reflexes were absent bilaterally in the lower limbs and there was decreased sensation to light touch. Tissue cultures taken from the affected vertebrae were all negative at this time, and given the patient’s debilitated status, reconstructive spine surgery was indicated.

### Second surgical operation

**Fig. 1:**
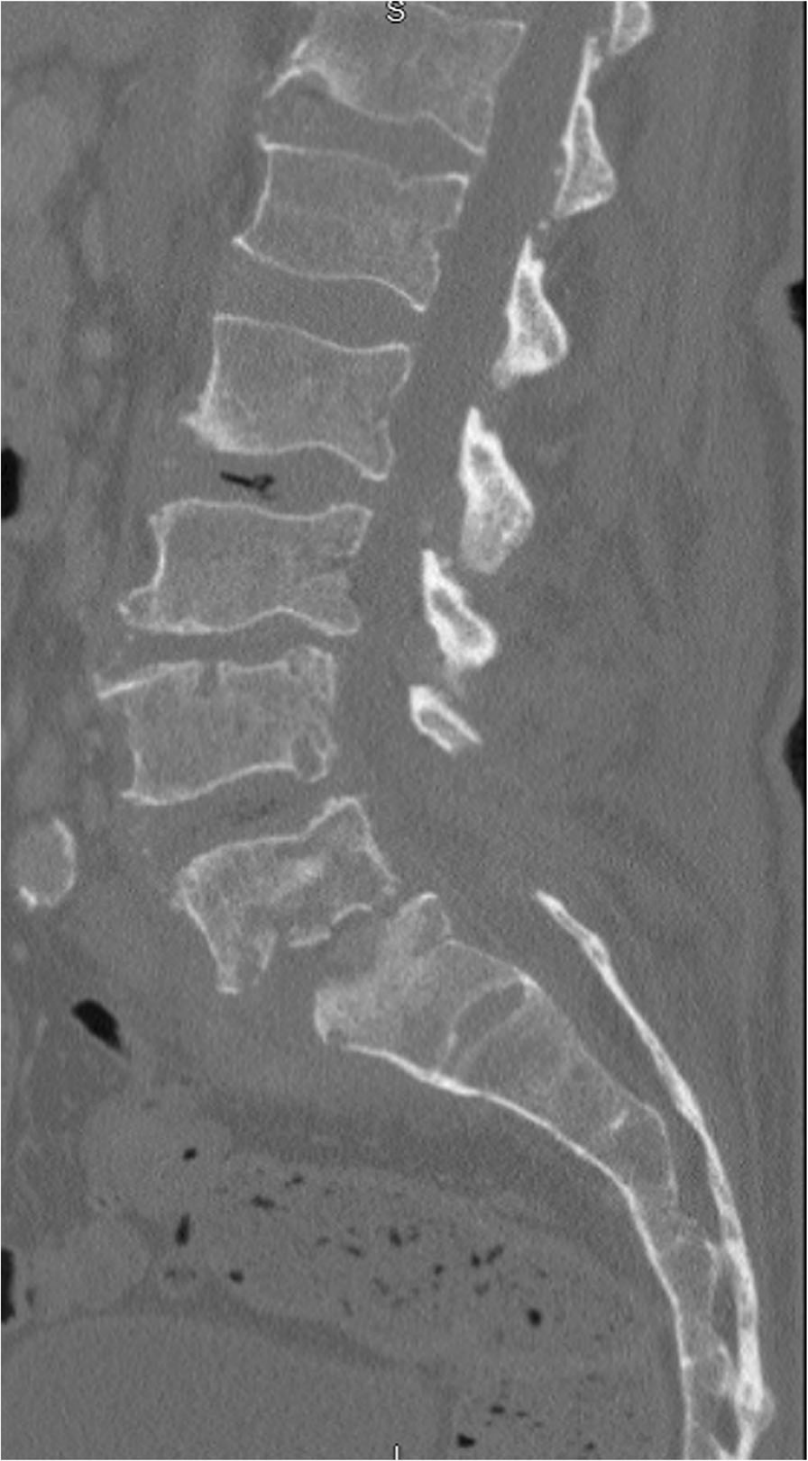
Lumbar CT Sagittal Reconstruction: progression of osteomyelitis at L4 and L5 with progressed anterolisthesis of L5

**Fig. 2:**
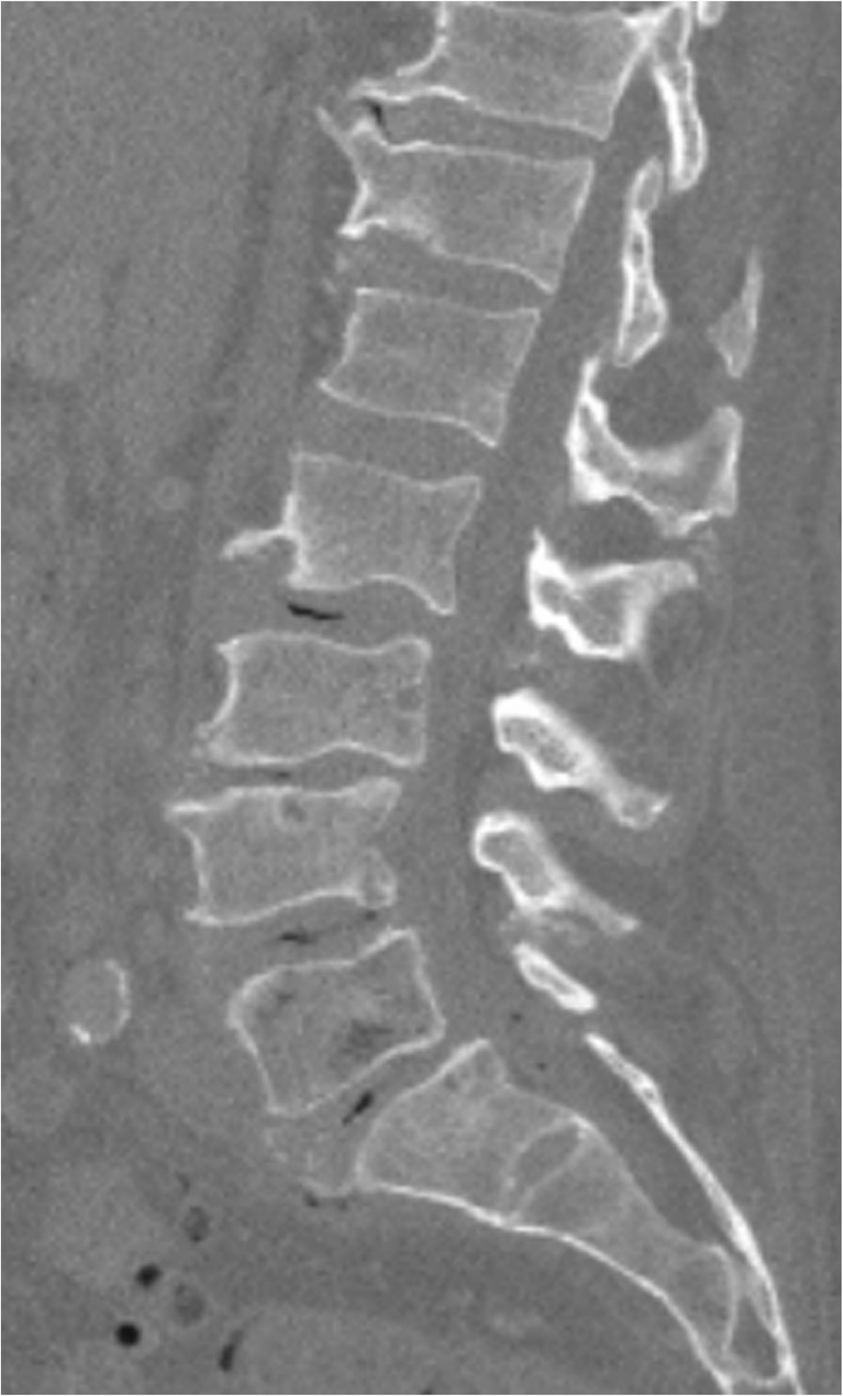
Lumbar CT Sagittal reconstruction: L5 with early osteomyelitic change

**Fig. 3:**
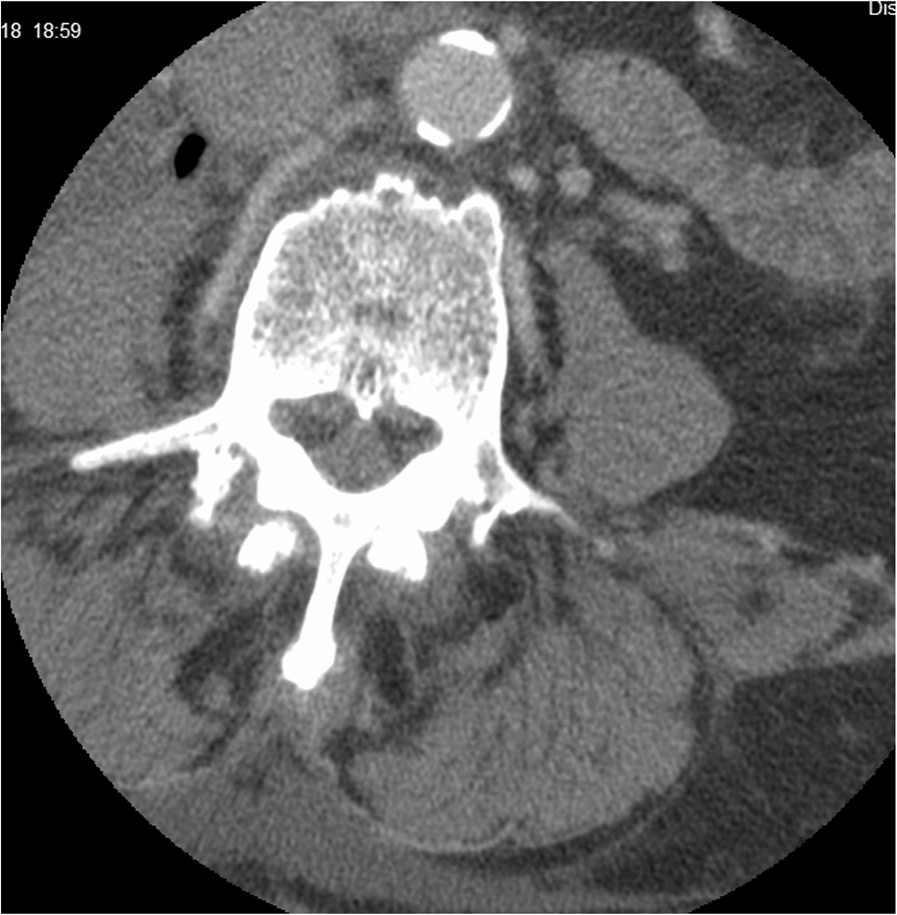
Axial CT lumbar spine: note air within the abscess in the right iliopsoas muscle

**Fig. 4:**
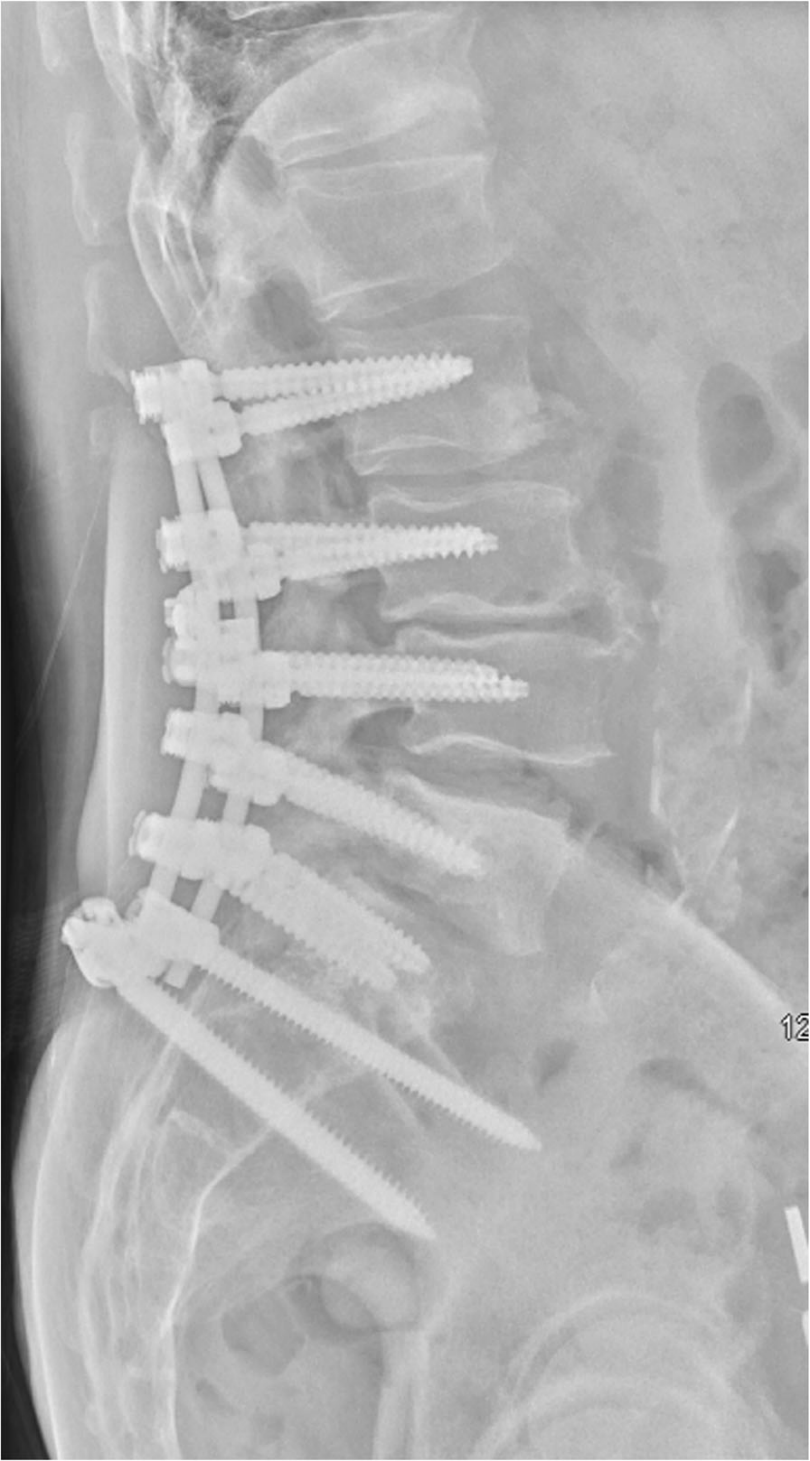
Lateral Lumbar X-ray: L2-Pelvis fusion construct with maintenance of lordotic alignment

**Fig. 5:**
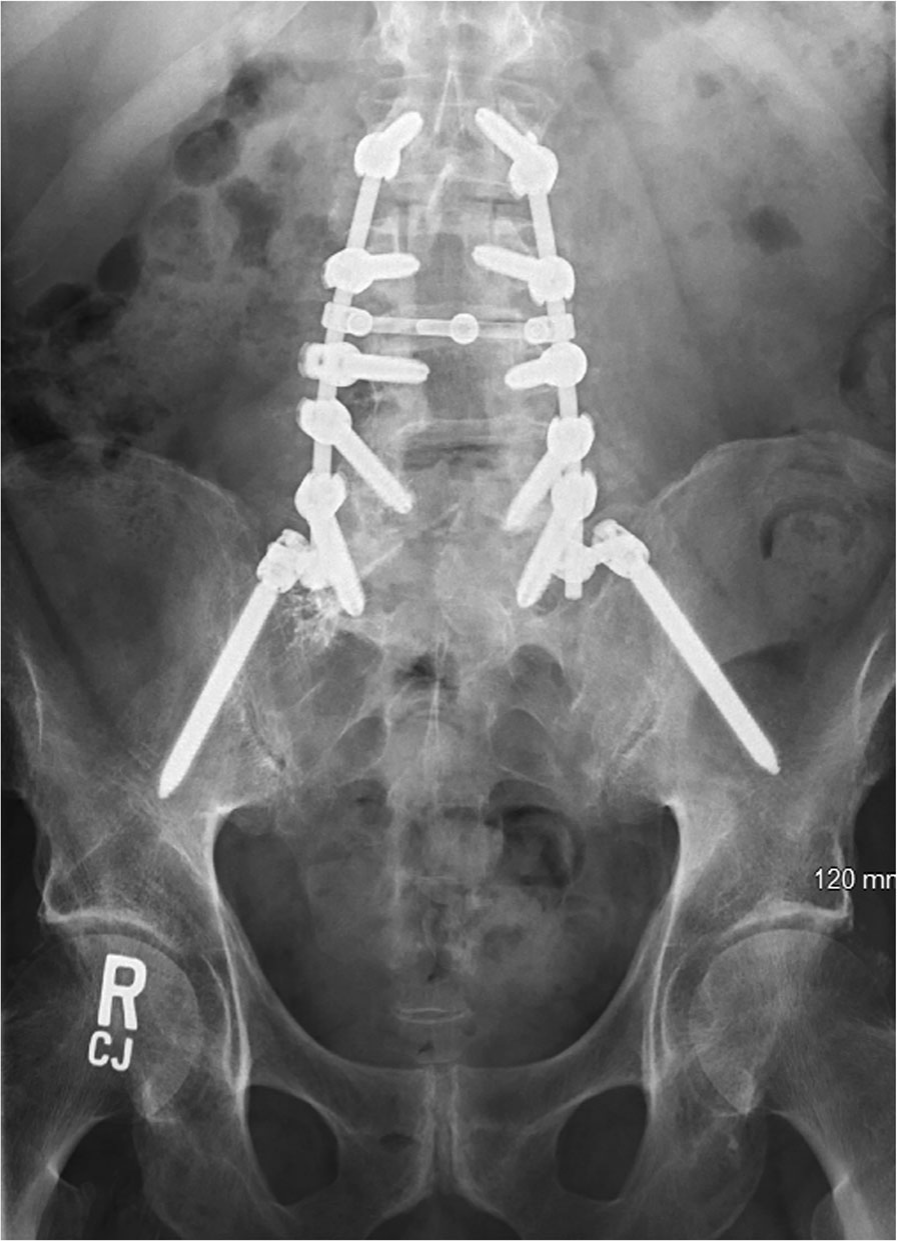
AP Lumbar X-ray: L2-Pelvis fusion construct

Preoperative X-ray revealed spondylolisthesis of L5 on S1, and a CT myelogram showed severe multilevel spinal stenosis from L2 to S1. Therefore, the patient underwent a L2-S1 lumbar laminectomy with posterior segmental instrumentation and posterolateral arthrodesis from the L2 to the ilium using pedicle screws and iliac bolts bilaterally. Decompression of spinal nerves was achieved by posterior lumbar laminectomy, bilateral medial facetectomies, and bilateral foraminotomies at all spinal levels between L2 and S1. Given the patient’s history of infection, an allograft was used to form a final instrumented fusion mass. (Figs. 1, 2, 3, 4 and 5).

### Post-operation

The patient recovered well after surgery. Final blood and tissue cultures were all negative. CT scans indicated resolution of the remaining abscesses. He was discharged and instructed to complete the previously prescribed course of antibiotics and follow-up with rehabilitation facilities. After 12 months of rehabilitation therapy, the patient regained a majority of mobility and ambulated with cane. However, at the 12 month follow up visit, he complained of groin pain. Additional physical therapy including aqua therapy was ordered and followed. At the 18 month follow up visit, the patient is noted to be doing quite well with occasional back discomfort and continues to ambulate with a cane.

## Discussion and conclusion

Here we present the case of an elderly male that suffered from vertebral osteomyelitis secondary to *Veillonella* bacteremia that resulted from a tooth abscess. To the best of our knowledge, we could identify only one report that attributes its case of vertebral *Veillonella* osteomyelitis to the oral cavity [9]. Additionally, there have been seven reports of vertebral *Veillonella* osteomyelitis and two reports of *Veillonella* foot osteomyelitis in diabetics. Other reported cases exist, but they could not identify an underlying source [9, 13].

*Veillonella* species are generally found in polymicrobial processes, and our case seems to adhere to this as no singular species could be identified. They have been implicated in dental infections, pulmonary infections, endocarditis, prosthetic joint infections, cardiac prosthetic valve infections and osteomyelitis [4, 7, 9, 10, 13]. None of these predisposing risk factors could be identified in our patient. These bacteria are typically resistant to vancomycin, tetracycline, aminoglycosides, and ciprofloxacin, but infections are traditionally known to respond well to therapy with penicillin [7, 13]. In vitro, *Veillonella* is usually susceptible to cephalosporins, clindamycin, metronidazole, and chloramphenicol. Due to the scarce number of reports on *Veillonella* as a pathogen associated with invasive infection, there are no clear treatment recommendations in the literature. A few reports point to penicillin, cephalosporins, chloramphenicol, clindamycin, and metronidazole as providing positive responses [7, 13–18].

In our unique case, the patient’s allergies to penicillin and cephalosporin complicated his treatment course as extra steps were required to initially desensitize him to penicillin. For the first treatment course, the patient was given penicillin followed by a combination of metronidazole and fluoroquinolone for a total of 6 weeks. However, during this modified treatment period, the *Veillonella* species seemed to have invaded even more of the vertebral areas, despite the patient’s initial spinal surgery to limit spread of infection and decompress the spinal nerves. This led to worsening symptoms, recurrent visits to the hospital, and continued persistence of *Veillonella* in blood and tissue cultures of the vertebrae. The patient was given a second course of treatment consisting of vancomycin, metronidazole, and fluoroquinolone. Two weeks into treatment, blood cultures and biopsy cultures of the bone tissue were all negative. Thus, posterior lumbar laminectomy and segmental instrumentation was performed.

It is strongly believed that the source of *Veillonella* infection in this patient was the oral cavity. Examination of the patient’s past medical history revealed a tooth abscess which was incised and drained by an endodontist six weeks before the initial presentation. Due to the patient’s travel plans, antibiotics were prescribed only after the first procedure. We believe that the delay in the onset of antibiotic treatment is what led to the initial bacteremia that ultimately took root in the lower lumbar vertebrae.

## Data Availability

Not Applicable.

## Abbreviations

AP: Anteroposterior
CT: Computed Tomography
IV: Intravenous
MICU: Medical Intensive Care Unit
MRI: Magnetic Resonance Imaging
PICC: Peripherally Inserted Central Catheter
